# Dynamic viscosity measurement in non-Newtonian graphite nanofluids

**DOI:** 10.1186/1556-276X-7-360

**Published:** 2012-07-02

**Authors:** Fei Duan, Ting Foong Wong, Alexandru Crivoi

**Affiliations:** 1 School of Mechanical and Aerospace Engineering, Nanyang Technological University, 639798, Singapore

**Keywords:** Dynamic viscosity, Graphite nanofluids, Nanoparticle aggregation, Non-Newtonian flow

## Abstract

The effective dynamic viscosity was measured in the graphite water-based nanofluids. The shear thinning non-Newtonian behavior is observed in the measurement. On the basis of the best fitting of the experimental data, the viscosity at zero shear rate or at infinite shear rate is determined for each of the fluids. It is found that increases of the particle volume concentration and the holding time period of the nanofluids result in an enhancement of the effective dynamic viscosity. The maximum enhancement of the effective dynamic viscosity at infinite rate of shear is more than 24 times in the nanofluids held for 3 days with the volume concentration of 4% in comparison with the base fluid. A transmission electron microscope is applied to reveal the morphology of aggregated nanoparticles qualitatively. The large and irregular aggregation of the particles is found in the 3-day fluids in the drying samples. The Raman spectra are extended to characterize the *D* and *G* peaks of the graphite structure in the nanofluids. The increasing intensity of the *D* peak indicates the nanoparticle aggregation growing with the higher concentration and the longer holding time of the nanofluids. The experimental results suggest that the increase on effective dynamic viscosity of nanofluids is related to the graphite nanoparticle aggregation in the fluids.

## Background

Nanofluids, consisting of suspended nanoscale solid particles, can improve thermal conductivity and heat transfer coefficient from the base fluids [1-10]. The effectiveness of thermal property enhancement of nanofluids depends on nanoparticle amount, particle size, particle materials, particle shape, base fluids, etc. However, since nanofluids are suspensions with nanoparticles in their base fluids, achieving a stable dispersion in nanofluids would benefit industrial applications. Nanoparticles are expected to stabilize the fluids more effectively than the microparticles, the fluid properties including the dynamic viscosity would change accordingly. The viscosity of nanofluids is important for nanofluid transport related to flow dynamics and heat transfer. The spherical shaped Al_2_O_3_, TiO_2_, and the other nanoparticles have been widely studied in the nanofluids [2-5,8]. The results showed an enhancement on the effective dynamic viscosity as an increase of concentrations. A strong correlation was indicated between the rheological behavior and the structure of nanoparticles in the nanofluids. The Al_2_O_3_-water nanofluid exhibited as a Newtonian flow after freshly prepared, but a shear thinning non-Newtonian flow after the aggregation was formed in the nanofluids. The dynamic viscosity had a significant increase as a result. However, the properties can be resumed after the re-ultrasonication process, in which the aggregates were dispersed again [11]. The main nonspherical nanoparticles in suspensions under the study are carbon nanotubes (CNTs) and graphite on thermal conductivity [1,9,10]. However, there are limited reports on the nanofluid dynamic viscosity with the nanoparticles at different heights to width aspect ratios, especially for the graphite nanoparticles. Ding et al. measured the dynamic viscosity of CNTs-water nanofluids as a function of shear rate, showing the fluids with a non-Newtonian property [10]. The viscosity of the nanofluids was found to increase with the increasing concentrations of CNTs and the decreasing temperature. Yang et al. investigated the rheological behavior of poly(*α*-olefin) solutions dispersed by the rodlike CNTs with an aspect ratio of about 30, or the disklike graphite nanoparticles an aspect ratio of about 0.025 [9]. The nanofluids acted as a shear thinning fluids. The above studies suggest that the nanoparticles may aggregate in the base fluids, and the aggregation would affect the rheological properties. To explicate the phenomena, the investigation of the dynamic viscosity is carried out in the graphite-water nanofluids for the potential application. The morphology of the nanoparticle aggregates and the structure in molecular vibration are demonstrated in this paper by using a transmission electron microscope and a Raman spectroscope, respectively.

## Methods

In the experiments, a two-step method was used to prepare the graphite water-based nanofluids. The graphite nanoparticles were supplied by SkySpring Nanomaterials, Inc. (Houston, TX, USA) with a reported average size of 3 to 4 nm. We dispersed the nanoparticles in the 40 mL deionized water to prepare the nanofluids with the volume concentrations at 1 %, 2 %, 3 %, and 4 % without adding any surfactant in order to study the effect of nanoparticle aggregation. The next step for the nanofluids was to undergo a mechanical stirring process with a magnetic stirrer at a rotation speed 540 rpm for about 7 h. Then, the nanofluids were performed under ultrasonication by using the ultrasonic bath (Elmasonic E 15H, Singen, Germany) continuously for about 1.5 h to prevent the well-dispersed fluids from aggregation initially.

The effective dynamic viscosity of the graphite-water nanofluids was measured directly with a standard controlled shear rate rheometer (Contraves LS 40, Mettler-Toledo, Greifensee, Switzerland) which has a cup and bob geometry. This instrument requires only a volume of liquid of approximately 5 mL. The instrument was calibrated by measuring the dynamic viscosity of the deionized water. The calibration results showed the measurement error within ±1% from the viscosity value of 0.000891 Pa·s. All measurements in this study were performed at 1 atm and 298.15 *K*. The effective dynamic viscosity of the nanofluids was measured instantly after the ultrasonication agitation. Thereafter, the same nanofluids were measured again after 3 days, which is determined by the experimental observation with the stratified fluids. Before the measurement, the fluids were shaken to prevent the possible particle sediment in the measurement. The relative effective dynamic viscosity is calculated with a reference value of the base fluid (pure water).

A transmission electron microscope (TEM, JEOL, JEM-2010, Tokyo, Japan) is applied to reveal the microstructural morphology of the graphite particles in the dried samples from the nanofluids. In preparation, the nanofluids in the volume fraction of 1% were diluted so as to reduce the possibility of the particle agglomeration in preparing the TEM samples in the drying process [12]. Then, a little drop of the nanofluid samples was dried naturally by placing on the copper grid coated with carbon film. The TEM instrument was used at an operating voltage of 200 kV in the graphite-water nanofluids instantly after preparation (fresh) and 3 days after, respectively. The Raman spectra were to disclose the molecular structure of materials. A Renishaw inVia Raman spectroscope (Wotton-under-Edge, UK) was applied by using the 514 nm He-Ne laser source with a laser power setting at 10 mW to determine the structure of nanoparticle aggregation on the basis of the molecular vibration. The fresh nanofluids and the fluids held for 3 days were sampled and then measured at room temperature for the Raman spectra.

## Results and discussion

**Figure 1:**
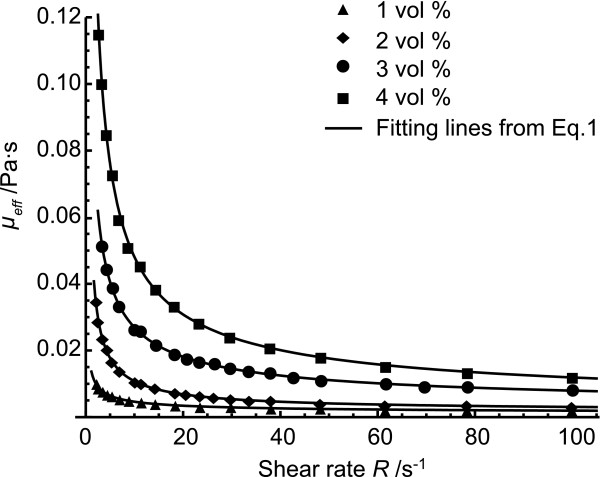
The effective dynamic viscosity as a function of steady shear rate in the fresh nanofluids.

The steady shear measurement was conducted at room temperature (298.15 *K*) for the series of nanofluids with the volume concentrations at 1%, 2%, 3%, and 4%. The effective dynamic viscosity of the fresh nanofluids, which were just prepared, is shown in Figure 1. It can be seen that the effective dynamic viscosity decreases with an increase of the shear rate in the nanofluids for a given concentration. The viscosity increases with the increasing loading of nanoparticles at the same shear rate, the effective dynamic viscosity has a higher value at 4 vol% than that at 1%. The dispersions with the graphite particles are shear thinning at low shear rates and approach a constant dynamic viscosity at high shear rates. The nanofluids act as the non-Newtonian flows.

With the assumption of a pseudoplastic flow, the modified Cross model [13], expressed in Equation 1, is applied to fit the experimental data,

(1) $$\frac{\mu_{\text{eff}}-\mu_{\infty }}{\mu_{0}-\mu_{\infty }}=\frac{1}{1+\alpha R^{n}}$$

where *R* is the shear rate, *μ*_0_ is the dynamic viscosity at zero rate of shear, $\mu_{\infty }$ is the dynamic viscosity at infinite rate of shear, *α*and *n* are constant. The fitting parameters are listed in Table 1. The effective viscosities, $\mu_{\infty }$ and *μ*_0_, increase with an increase of the volume concentration from 1% to 4%. The dynamic viscosity at infinite shear rate of the 4 vol% nanofluids is over 2.68 times that of the 1 vol% nanofluids.

**Table 1:** Fitting parameters of the steady shear measurement for the nanofluids held 3 days (*nff*)

**Experiment:**	***μ*_0_/Pa*·*s**	$\mu_{\infty }$**/Pa** ***·*** **s**	***α***	***n***
*nff*1	5.02911×10^10^	0.000968736	3.64600×10^12^	0.577278
*nff*2	1.07711×10^11^	0.00165829	1.81188×10^12^	0.814235
*nff*3	4.85370×10^11^	0.00218935	4.57560×10^12^	0.632872
*nff*4	1.11869×10^13^	0.00259891	5.03442×10^13^	0.689077

**Figure 2:**
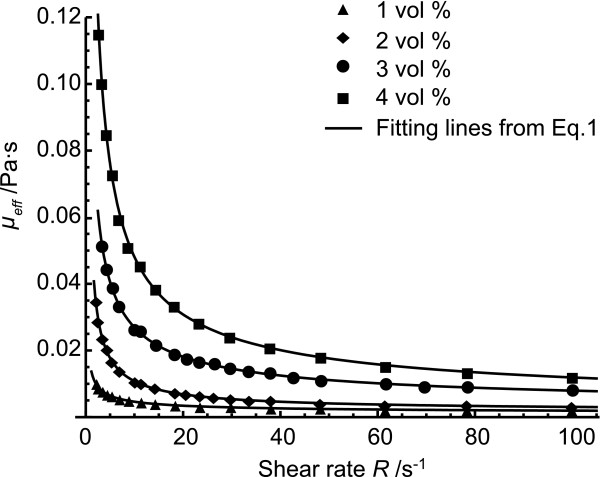
The effective dynamic viscosity as a function of steady shear rate in the 3-day fluids.

Similarly, the effective dynamic viscosity of the nanofluids held for 3 days is shown with a non-Newtonian behavior as well, illustrated in Figure 2. The effective dynamic viscosity decreases dramatically under low shear rates and approaches a constant dynamic viscosity at high shear rates. At a given shear rate, the dynamic viscosity increases with the increasing loading of particles, similar to that in the fresh nanofluids. However, the enhancement of the effective dynamic viscosity is much higher than the fresh nanofluids under the same volume concentration. The experimental data were also fitted to obtain the parameters of *μ*_0_, $\mu_{\infty }$, *α*, and *n*, listed in Table 2. The dynamic viscosity at infinite shear rate, $\mu_{\infty }$ increases to 1.34 times for the 2 vol% of 3-day fluids, 3.39 times at 3 vol%, 11.21 times at 4 vol% in comparison with the 1 vol% nanofluids three days after the preparation.

**Table 2:** Fitting parameters of the steady shear measurement for the nanofluids held 3 days (*nfo*)

**Experiment:**	***μ*_0_/Pa*·*s**	$\mu_{\infty }$**/Pa*·*s**	***α***	***n***
*nfo*1	6.78665×10^11^	0.00197601	1.28804×10^13^	0.786853
*nfo*2	3.76320×10^12^	0.00265571	4.77138×10^13^	0.664450
*nfo*3	1.17756×10^13^	0.00670576	4.46724×10^13^	0.679144
*nfo*4	5.93012×10^13^	0.02214670	1.01070×10^14^	0.826530

The fitting errors were analyzed for each of the effective dynamic viscosity measurements. The average fitting errors were calculated from Equations 2 and 3. The mean of absolute fitting errors and the absolute average fitting error are listed in Table 3.

(2) $${\overset{—}{\left(\frac{\Delta \mu }{\mu }\right)}}_{\mathrm{MA},j}=\frac{\sum |(\mu_{\text{fit}}-\mu_{\text{eff}})/\mu_{\text{eff}}|}{k}$$

where ${\overset{—}{\left(\frac{\Delta \mu }{\mu }\right)}}_{\mathrm{MA}}$ is the average of absolute fitting errors, *MA*, of the effective dynamic viscosity of the nanofluids, *j* is for the fresh nanofluids (*nff *) and or the 3-day fluids (*nfo*), *μ*_*fit*_ is the fitted dynamic viscosity at the measured rate of shear, *μ*_*eff*_is the measured dynamic viscosity, and *k* is the number of readings at a run of viscosity measurement.

(3) $${\overset{—}{\left(\frac{\Delta \mu }{\mu }\right)}}_{\text{AM,j}}=|\frac{\sum (\mu_{\text{fit}}-\mu_{\text{eff}})/\mu_{\text{eff}}}{k}|$$

where ${\overset{—}{\left(\frac{\Delta \mu }{\mu }\right)}}_{\mathrm{AM}}$ is the absolute average fitting error, *AM*.

**Table 3:** The fitting errors of the effective dynamic viscosity of fresh nanofluids and 3-day fluids

	**1%**	**2%**	**3%**	**4%**
${\overset{—}{\left(\frac{\Delta \mu }{\mu }\right)}}_{\text{MA,nff}}$	0.49%	0.83%	1.23%	1.28%
${\overset{—}{\left(\frac{\Delta \mu }{\mu }\right)}}_{\text{AM,nff}}$	0.00%	0.02%	0.05%	0.11%
${\overset{—}{\left(\frac{\Delta \mu }{\mu }\right)}}_{\text{MA,nfo}}$	0.98%	1.27%	1.33%	1.87%
${\overset{—}{\left(\frac{\Delta \mu }{\mu }\right)}}_{\text{AM,nfo}}$	0.04%	0.22%	0.24%	0.08%

As listed in Table 3, the average of absolute fitting errors is 0.49% for the 1 vol% fresh graphite-water nanofluids compared with the value at 0.98% in the 1% nanofluids held for 3 days. For a higher volume concentration at 4%, the mean of absolute fitting errors is 1.28% for the fresh nanofluids, but 1.87% for the 3-day fluids. The absolute average fitting error has a smaller value compared with the mean of the absolute fitting errors in the same nanofluids. The maximum absolute average fitting error is 0.11% for the fresh nanofluids while it is 0.24% for the 3-day fluids. It can be seen that the fitting curve is very close to the experimental data.

**Figure 3:**
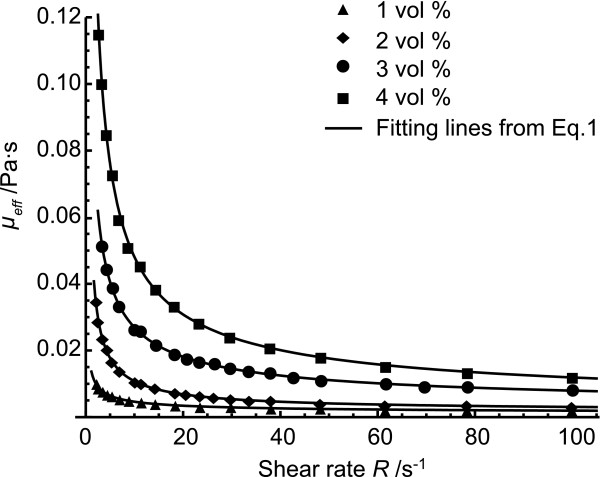
**The relative dynamic viscosity,**$\mu_{\infty }/\mu_{f}$**, as a function of particle volume concentration.**

Figure 3 shows the relative effective dynamic viscosity, $\mu_{\infty }/\mu_{f}$, as a function of the nanoparticle volume concentration, in which *μ*_*f*_is the dynamic viscosity of the base fluid. It is found that the viscosity ratio increases monotonically as a function of the volume concentration for both the fresh nanofluids and the 3-day fluids. The relative effective dynamic viscosity at infinite shear rate increases gradually to 2.92 in the fresh nanofluids, but up to 24.86 in the nanofluids held for 3 days from 1% to 4% in volume fraction. Yang et al. showed a similar trend in the nanofluids of graphite and poly(*α*-olefin) solutions, the relative effective viscosity increased from 1.15 at the volume fraction at 0.39% to 1.33 at 0.78% [9]. As seen from Figure 3, the relative effective dynamic viscosity shows a significant higher value in the 3-day fluids than that in the fresh nanofluids under the same concentration at the volume fraction of 1%, 2%, 3%, or 4%, respectively. The relative effective dynamic viscosity at infinite shear rate is 1.09 for the fresh nanofluids at the volume fraction at 1%, but 2.22 for the 1% 3-day fluids. As shown in the insert of 3, the increasing dynamic viscosity gradient reported by Yang et al. [9] is shown in between the fresh nanofluids and the fluids held for three days in this study. The enhancement can be mainly explained that a higher aggregation of nanoparticles, induced by a higher concentration, results in a higher effective dynamic viscosity [11]. Even though the nanofluids held for 3 days were remixed before the measurement, the shaking process cannot totally break down the aggregation formed with the time.

Similarly, Kim et al. reported that the CNT-based nanofluids had such the phenomenon by pointing out that the high surface effect and the strong van der Waals force drive the nanoparticles to form the aggregation in the suspensions [14]. Since most of aggregates might be destroyed under high shear rates, the nanofluids are shown in shear thinning non-Newtonian behaviors [9,11,15]. We have to mention that the graphite-water nanofluids have a different flow property from the Al_2_O_3_-water nanofluids, which are Newtonian flows if the nanofluids are freshly prepared, but non-Newtonian flows if the fluids have large aggregates [11]. The different results might be from the various nanoparticle sizes, species, and configuration. The graphite-water nanofluids might have the particle aggregation just after the preparation, and show the non-Newtonian flow properties. The size of aggregation in nanofluids would increase with the increase of the particle volume concentration and the holding time. Thus, it would take a higher force to break the ligand structure among particles in the aggregated fluids [9,11], as a result, a high effective dynamic viscosity ratio can be observed in Figure 3.

**Figure 4:**
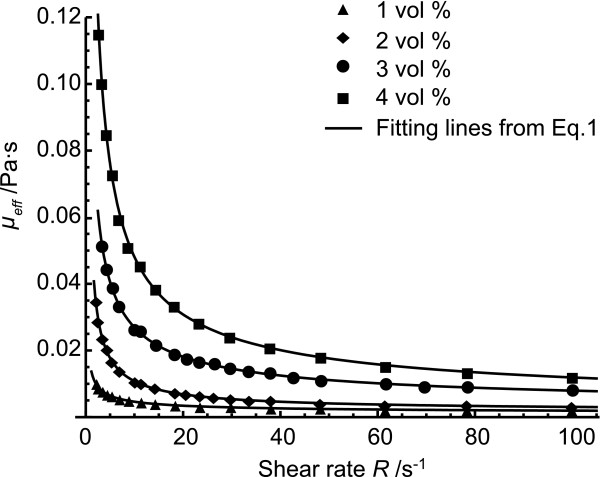
TEM images of graphite nanoparticle aggregation from the fresh nanofluids (a) and 3-day fluids (b).

The effective dynamic viscosity enhancement can also be qualitatively explained with the viscosity ratio between a nanofluid (*nf*) and its base fluid (*f *) in the model [16], $\frac{\mu_{\mathrm{nf}}}{\mu_{f}}={\left(1-\frac{\phi_{a}}{\phi_{m}}\right)}^{-[\mu ]\phi_{m}}$, in which *μ* is the intrinsic viscosity for spherical particles with a value of 2.5, *ϕ*_*a*_ is the volume fraction of aggregates, and *ϕ*_*m*_is the volume fraction of densely packed spheres. The volume fraction of the aggregates can be expressed as $\phi_{a}=\phi {\left(\frac{d_{a}}{d}\right)}^{3-d_{f}}$, in which *d*_*f*_is the fractal dimension of the aggregates. When the nanoparticle aggregation size, *d*_*a*_, increases, the magnitude of $\frac{d_{a}}{d}$ increases. Thus, the volume fraction of the aggregates increases, and the viscosity ratio increases based on the model of Krieger and Dougherty [16]. As shown in the microstructure in Figure 4, the shape of graphite nanoparticles is not spherical, and the aggregates of particles are more complex in the 3-day fluids. The intrinsic viscosity, *μ*, consequently changes larger with the complicated shape [17]. This could also account for the rise in the effective dynamic viscosity as the volume concentration or the holding time increases. The relative effective viscosity of the 3-day fluids at the volume concentration of 4% is as 22.86 times as the value of the fresh nanofluids at 1 vol%, as shown in Figure 3.

The TEM images of the graphite particles dried from the nanofluids are shown in Figure 4. It is found that the average diameter of graphite particles dispersed in the nanofluids just after the ultrasonic agitation is up to 50 nm shown in Figure 4a. The nanoparticles are still larger than those specified by the supplier in the powder form. It suggests that the graphite nanoparticles have aggregated into a certain size even in the fresh nanofluids, resulted from the high surface effect of nanoparticles and the inter-particle attraction [11,18]. However, the graphite particles were significantly aggregated if the nanofluids were held for 3 days, as shown in Figure 4b. The size of aggregated graphite particles is larger than 150 nm at least. The highly fragmented aggregation clusters were found in the microstructure analysis. The aggregation of nanoparticles in the graphite-water nanofluids increased with a longer holding time. It supports the aforementioned discussion that the effective dynamic viscosity increases at the 3-day fluids. Note that the nanoparticle boundary is detectable in the aggregates shown in Figure 4a. We can estimate that the largest dimension of the particle is at about 18 nm. Thus, if the 3 to 4 nm is treated as the thickness of the graphite nanoparticle, the height to width aspect ratio could be up to 0.17, which is much larger than those used by Yang et al. [9].

**Figure 5:**
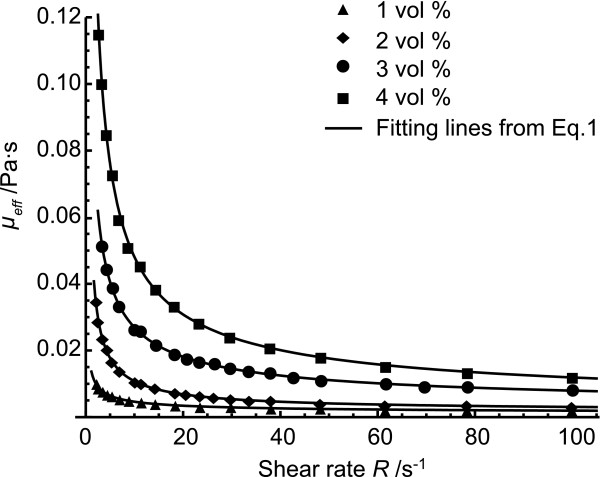
Raman spectra of the fresh nanofluids and 3-day fluids.

Figure 5 illustrates the Raman spectra at various volume concentrations from 1% to 4% for the fresh nanofluids and the 3-day fluids. Two characteristic peaks are observed in the graphite-based nanofluids, locating in the range of 1,570 to 1,594 cm^−1^ and 1,330 to 1,360 cm^−1^. These features could be characterized as the *G* peak and the *D* peak [19-21], marked in Figure 5. In the crystallization analysis, the *G* peak of graphite at 1,575 cm^−1^indicates the *s*^*p*2^vibration of the carbon atoms in the structure, and the *D* peak of graphite at 1,355 cm^−1^, suggesting the *s*^*p*3^hybridization of carbon atoms, is resulted from defects, disorder, and impurities in the materials. As illustrated in Figure 5, the intensities of the *D* and *G* peaks increase as an increase of the volume concentration in the fresh nanofluids, the strength of the *D* peaks increases to about 10 times as the volume concentration is 2%, and 20 times as the concentration is 4%. Then, the intensities acutely increase with an increase of the particle volume concentration in the 3-day fluids. A dramatic increase of the intensity at the *D* peak is found between the fresh nanofluids and those held for 3 days for a given concentration, i.e., the intensity increases over 70 times in the nanofluids with 1 vol%. The other three increase the intensity of the *D* peak above 100 times compared with the 1% fresh nanofluids. It is indicated that the effective intensity of the *D* peak in the nanofluids qualitatively reflects the size of the aggregation clusters of graphite nanoparticles in the nanofluids. The size of clusters became larger at a higher concentration and a longer holding time. The results from the Raman spectra are consistent with the dynamic viscosity measurements and the TEM microstructures. In addition, the intensity ratio at about a unit between the *D* peak and the *G* peak also suggests the cluster and chain groups formation [22].

## Conclusions

The effective dynamic viscosity of the graphite-water nanofluids is experimentally found to decrease with an increase of shear rate in a given particle volume fraction (Figures 1 and 2). The nanofluids act as the shear thinning non-Newtonian flows. The data of the effective dynamic viscosity in nanofluids are fitted numerically, the relative effective dynamic viscosity at infinite rate of shear increases to 2.92 in the fresh nanofluids at 4 vol% in comparison of the base fluid, but 24.86 for the nanofluids held for 3 days (Figure 3). The microstructure of the diluted nanofluids indicates that the aggregation of nanoparticles is significantly higher in the 3-day fluids than that in the fresh nanofluids, as shown in Figure 4. The Raman spectra are used for showing the formation of larger graphite nanoparticle aggregation with an increase of the volume concentration or the holding time of the nanofluids in Figure 5. This study suggests that the aggregation would happen in the nanofluids which have not been treated specially by adding the surfactant, controlling the pH value, etc. The aggregation would dramatically change the nanofluid properties including viscosity consequently.

## Competing interests

The authors declare that they have no competing interests.

## Authors’ contributions

TFW carried out the viscosity measurement of nanofluids and participated in the manuscript writing. AC participated in analyzing of the data. FD initiated the study and revised the manuscript. All authors read and approved the final manuscript.

## Authors’ informations

FD is an assistant professor on thermofluids at Nanyang Technological University. TFW was an undergraduate student for his final year project under FD. AC is a Ph.D. student on nanofluids.
